# USING TELEPRESENCE ROBOTS TO SUPPORT FAMILY VIRTUAL VISITS DURING THE COVID-19 PANDEMIC

**DOI:** 10.1093/geroni/igac059.270

**Published:** 2022-12-20

**Authors:** Lillian Hung

**Affiliations:** University of British Columbia, Vancouver, British Columbia, Canada

## Abstract

In this project, virtual family visits were made to residents of four Canadian long-term care homes using a telepresence robot. Guided by the Consolidated Framework of implementation Research (CFIR), we conducted an online survey, interviews, focus groups, and observations to explore the participants’ experience. Analysis identified three themes: (a) relative advantage (easy to visit), (b) capacity for change (readiness and organizational support), and (c) cultural safety (champions leading the way during challenging times). Preliminary results indicate that staff, residents, and families accepted the robot for easy connection but had concerns about privacy issues. Future research should apply inclusive methods to bring relevant stakeholders together to explore user experiences fully: who is affected in what ways and the benefits, risks, and burdens of emerging technologies.

